# Structural insights into leishmanolysins encoded on chromosome 10 of *Leishmania (Viannia) braziliensis*

**DOI:** 10.1590/0074-02760160522

**Published:** 2017-09

**Authors:** Amanda Sutter, Deborah Antunes, Mariana Silva-Almeida, Maurício Garcia de Souza Costa, Ernesto Raul Caffarena

**Affiliations:** 1 Grupo de Biofísica Computacional e Modelagem Molecular, Programa de Computação Científica, Rio de Janeiro, RJ, Brasil; 2 Fundação Oswaldo Cruz-Fiocruz, Instituto Oswaldo Cruz, Laboratório de Biologia Molecular e Doenças Endêmicas, Rio de Janeiro, RJ, Brasil

**Keywords:** *Leishmania* spp, Leishmania (Viannia) braziliensis, metalloproteases, leishmanolysins, comparative modeling, molecular dynamics

## Abstract

**BACKGROUND:**

Leishmanolysins have been described as important parasite virulence factors because of their roles in the infection of promastigotes and resistance to host’s defenses. *Leishmania (Viannia) braziliensis* contains several leishmanolysin genes in its genome, especially in chromosome 10. However, the functional impact of such diversity is not understood, but may be attributed partially to the lack of structural data for proteins from this parasite.

**OBJECTIVES:**

This works aims to compare leishmanolysin sequences from *L. (V.) braziliensis* and to understand how the diversity impacts in their structural and dynamic features.

**METHODS:**

Leishmanolysin sequences were retrieved from GeneDB. Subsequently, 3D models were built using comparative modeling methods and their dynamical behavior was studied using molecular dynamic simulations.

**FINDINGS:**

We identified three subgroups of leishmanolysins according to sequence variations. These differences directly affect the electrostatic properties of leishmanolysins and the geometry of their active sites. We identified two levels of structural heterogeneity that might be related to the ability of promastigotes to interact with a broad range of substrates.

**MAIN CONCLUSION:**

Altogether, the structural plasticity of leishmanolysins may constitute an important evolutionary adaptation rarely explored when considering the virulence of *L. (V.) braziliensis* parasites.

Leishmaniases are endemic parasitic diseases spread worldwide, mainly found in tropical and subtropical areas and southern Europe. Current reports indicate ~1.3 million of new cases annually, leading to approximately 20.000 deaths (WHO 2016). The clinical spectrum of leishmaniases comprises three forms: visceral, cutaneous and mucocutaneous. They are caused by parasites from the genus *Leishmania* transmitted by the bite of the insect vectors. Upon transmission to mammalian hosts, the promastigote form is phagocytosed by macrophages and transformed into amastigotes. While the cellular machinery of promastigotes evolved to optimize interactions with hosts, amastigotes are the main responsible for maintaining the infection (Teixeira et al. 2013).

Despite the progress attained on the understanding of the interaction between parasite and hosts, few advances in the treatment of leishmaniases were obtained. Moreover, there are several limitations associated with the administration of the currently available drugs, which are also highly toxic and may induce the development of resistant strains (Pereira et al. 2014). In this context, targeting the parasite virulence factors may constitute an essential step for the developing of more effective anti-*Leishmania* therapies with lower toxicity levels.

Nowadays, it is widely accepted that proteases from *Leishmania* spp exert a pivotal role in the parasite life cycle (Barrett 1994). Four classes of proteases have already been described in *Leishmania* parasites: serine, cysteine, aspartyl, and metalloproteases (Silva-Almeida et al. 2012), being 52% of the protease genes in the *L. (V.) braziliensis* genome corresponding to the last one (Silva-Almeida et al. 2014). However, it is still unclear how such diversity favors infection and survival of these parasites. *Leishmania* spp express abundant surface glycoproteins that are known as metalloproteases from the M8 family (subclan MA(M) - metzincins), also termed leishmanolysins (Barrett et al. 2012). These enzymes are the major protein component found on the surface of promastigotes, being highly active against polypeptides. They are membrane-bound metalloproteases (via GPI anchor), essential to parasite infection and protection from complement-mediated lysis (Joshi et al. 2002). The currently available leishmanolysin three-dimensional structure, (PDB ID: 1LML) (Schlagenhauf et al. 1998), revealed a three-domain structure (N-terminal, central region and C-terminal) containing the zinc-coordination motif HEXXH, as well as several conserved residues (~60%) in comparison to leishmanolysins from other species (e.g., *T. brucei*, *L. major*, *Crithidia*). This motif is similar to that commonly found in other metalloproteases from the metzincin class but differs from the defining motif HExxHxxGxxH due to a 62 a.a insertion region between the conserved glycine and the last histidine (Stöcker et al. 1995). However, an examination of the leishmanolysin structure revealed no disruption of the active site geometry in comparison to other metzincins (Schlagenhauf et al. 1998).

The chromosome 10 of *L. (V.) braziliensis* contains a large variety of leishmanolysin genes. To understand the molecular basis underlying this diversity, we attempted to characterise structurally several leishmanolysins encoded on this chromosome. We clustered 29 sequences in three distinct subgroups. Next, after structural modeling of these enzymes, we observed different distributions of the electrostatic potential on the surface of the insertion region containing the active site that correlate with the subgroups previously determined. Finally, we were able to characterize the microheterogeneity of the leishmanolysins active sites from molecular dynamics (MD) simulations. We also discuss the relevance of our findings in the context of the evolutionary adaptations displayed by *Leishmania* parasites.

## MATERIALS AND METHODS

*Sequence analysis* - We retrieved the *L. (V.) braziliensis* sequences corresponding to the metalloprotease family from the GeneDB (Silva-Almeida et al. 2014). Sequence similarity searches were performed using the BLASTp algorithm, and a multiple sequence alignment of the obtained sequences was carried out using T-Coffee Multiple Sequence Alignment Server (Notredame et al. 2000).

*Comparative modeling* - Candidate template structures for subsequent comparative modeling were taken from the PDB database (Berman et al. 2000). Three-dimensional models were generated with the Swiss-Model server (Arnold et al. 2006) employing as a template the high-resolution structure of *L. major* leishmanolysin (PDBid: 1LML) (Schlagenhauf et al. 1998), maintaining all input parameters as default. Three homology models were created for each submitted sequence and the structure displaying the highest QMEAN score (Benkert et al. 2008) was utilised for further analysis. The stereochemical quality of the models was evaluated with the structure analysis and verification server (SAVES) (Laskowski et al. 1993). Loop optimisations were performed with the KoBaMIN server (Rodrigues et al. 2012).

*MD simulations* - MD simulations were carried out using AMBER 14.0 in conjunction with the ff14SB force field (Case et al. 2014, Maier et al. 2015). Electrostatic interactions were treated using the particle mesh ewald (PME) algorithm with a cut-off of 10 Å. Each system was simulated under periodic boundary conditions in a cubic box filled with TIP3P water molecules (Jorgensen et al. 1983). To properly describe the coordination of the zinc ion within the active site, the protonation states of surrounding histidine residues were manually assigned: they were selected as Nd neutral tautomers. All systems were neutralised by adding counterions.

Subsequently, a two-step energy minimisation procedure was performed: (i) 2000 steps [1000 steepest descent (SD) + 1000 conjugate-gradient (CG)] with all heavy atoms harmonically restrained with a force constant of 5 kcal mol^-1^ Å^-2^; (ii) 5000 steps (2500 SD + 2500 CG) without position restraints. Next, initial atomic velocities were assigned using a Maxwell-Boltzmann distribution corresponding to an initial temperature of 20 K and the systems were gradually heated from 20 K to 310 K over one nanosecond utilising the Langevin thermostat. During this stage, all heavy atoms were harmonically restrained with a force constant of 10 kcal mol^-1^ Å^-2^. Systems were subsequently equilibrated during nine successive 100 ps equilibration simulations where position restraints approached zero progressively. After this period, the systems were simulated with no restraints at 310 K for 50 ns in the Gibbs ensemble with a pressure of 1 atm. Atomic coordinates and energies were recorded every 25 ps. The simulation trajectories were analysed using GROMACS package tools (Pronk et al. 2013).

*Electrostatic potential analysis and geometrical data from the active site* - We firstly performed a clustering analysis of the simulation trajectories with the module gmx cluster of the GROMACS package. In this step, we used the gromos algorithm (Daura et al. 1999) to identify conformational populations with an 1Å RMSD cut-off. The central structure corresponding to the most populated cluster in each simulation was submitted for subsequent calculations.

The electrostatic potential analysis was conducted with the APBS program (Baker et al. 2001). The AmberFF charge and radii parameters were assigned using the PDB2PQR server (Dolinsky et al. 2004). Subsequently, these structures were submitted to the 3V server (Voss & Gerstein 2010) to explore structural features of the active site (volume, surface area, sphericity, and effective radius). The characterization of the active site binding cavities was performed with KVFinder using default parameters (Oliveira et al. 2014).

## RESULTS

**Fig. 1:**
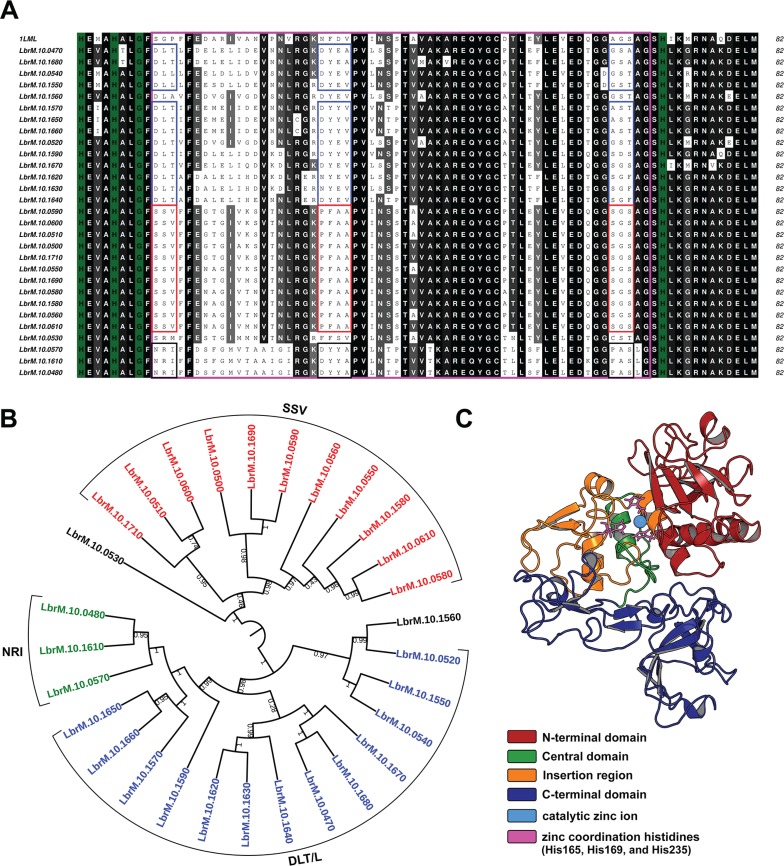
: (A) sequence alignment of the inserted regions of the 29 M8 metalloproteases encoded by chromosome 10 of *Leishmania (Viannia) braziliensis*. The sequence at the top (1LML) corresponds to the only leishmanolysin with the available crystal structure. The sequence alignment was performed using the ALINE program considering the insertion region (magenta box) of leishmanolysin class. Identical and similar residues are represented in black and gray, respectively. The three identified subgroups are colored in blue (DLT/L), red (SSV) and black (NRI); (B) dendogram profile of M8 metalloproteases encoded by chromosome 10 of *L. (V.) braziliensis*. The dendogram was built employing iTOL server using proteins sequences from geneDB. The numbers on the branches represent the evolutionary distance between different homologous proteins. The three motifs identified are colored and named near the sequence; (C) LbrM.10.0470 structure representing the three-dimensional form of this gene. The catalytic zinc ion is shown as a magenta CPK sphere. The zinc ligands are colored in orange and shown in ball-and-stick representation: His165, His169, and His235. The insertion region is colored orange and the central domain is represented in green. The N and C-terminal domains are represented in red and blue, respectively.

*Grouping leishmanolysins according to their insertion regions reveals three distinct subgroups* - From the multiple sequence alignment of the 29 leishmanolysin sequences encoded on chromosome 10, we distinguished subgroups presenting three specific patches of residues along the 62 a.a insertion region (Fig. 1A). The residue composition of the second and third patches is notably correlated with the first patch (Fig. 1A), suggesting that distant mutations may be evolutionary correlated. The two other patches were found 18 and 53 residues away from the first patch. From now on, we will refer to each particular group according to the initial triad of the first patch which starts one residue away from the conserved Gly 172 (Table). All of these regions present high identity with others of the same subgroup but low similarity with those from other subgroups.

**TABLE:** Patch and subgroups identified by sequence analysis of leishmanolysin sequences encoded on chromosome 10

			Patch 1			Patch 2				Patch 3			
						
	165	172	174			194				229			235
Subgroup 1 (DLX)	HEXXHXX									G	S	A	
						D	Y	E	A	G	S	T	
		GX	D	L	T	D	Y	E	V	A	S	T	H
			D	L	L	D	Y	Y	V	S	D	S	
						N	Y	E	V	S	G	S	
										S	G	F	
Subgroup 2 (SSV)	HEXXHXX	GX	S	S	V	P	F	A	A	S	G	S	H
Subgroup 3 (NRI)	HEXXHXX	GX	N	R	I	D	Y	Y	A	P	A	S	H
										F	A	S	

Clustering of these sequences evidenced that the most populated subgroup (n = 14) contained patches DLL, DLA and DLT, while the second subgroup (n = 12) encompassed sequences presenting SSV and SRM motifs. A third smaller subgroup of sequences, presenting the NRI patch (n = 3), was also found. Altogether, these analyses show considerable heterogeneity at the sequence level that allows the separation of leishmanolysins of *L. (V.) braziliensis* in subgroups (Fig. 1B).

*Structural heterogeneity on leishmanolysins evidences varying charge distributions in the active site* - To verify whether the leishmanolysin sequence diversity had an impact on their structural properties, we modeled them using comparative modeling. The same template was identified by BLASTp for all sequences: the leishmanolysin from *L. major* (PDBid: 1LML) (Schlagenhauf et al. 1998) using as selection criteria the maximum identity (59% ~ 71%) and coverage (50% ~ 97%). As observed in the crystal structure, the 62 a.a insertion is found adjacent to the active site and it represents most of the central domain (Fig. 1C).

All models were stereochemically validated, being more than 85% of residues located in the favorable regions. If we also consider the allowed regions, this number goes to 95% in all cases (Supplementary data, Table). Moreover, we obtained G-factors ranging from 0.04 to 0.37 among the models (values between 0 and 0.5 are considered acceptable). All QMEAN6 values obtained were higher than 0.6, therefore indicating high quality models.

**Fig. 2:**
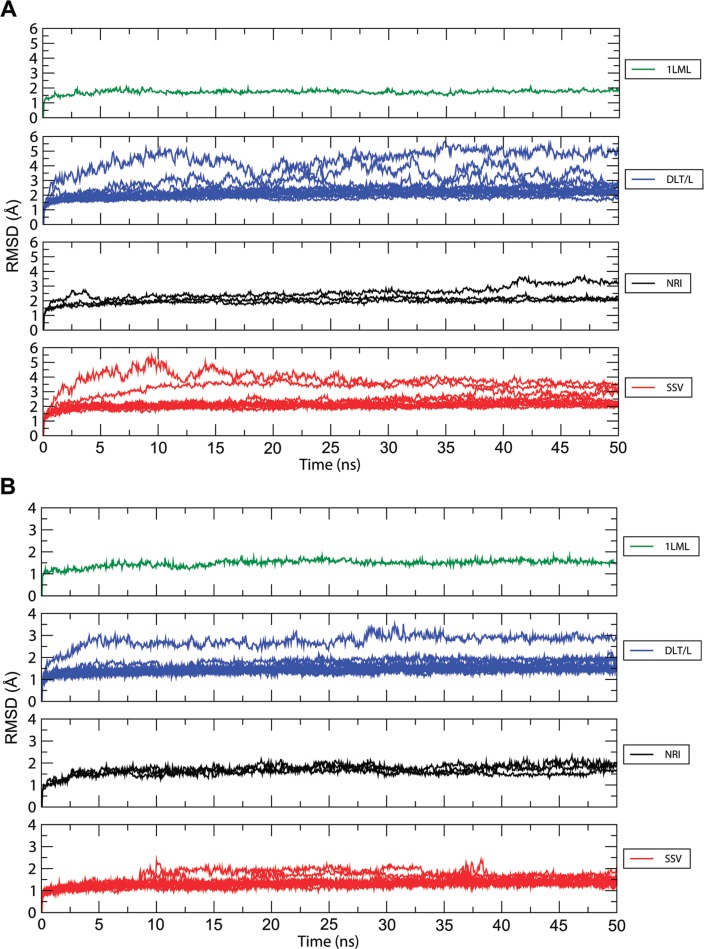
: backbone RMSD as a function of time for each simulated leishmanolysin considering: (A) the complete structure or (B) only the residues in the inserted region. The results corresponding to the simulation with the 1LML structure are represented in green. The curves corresponding to the simulations of the generated models belonging to each of the three subgroups (DLT/L, NRI, and SSV) are grouped in distinct plots and colored in blue, black and red, respectively.

**Fig. 3:**
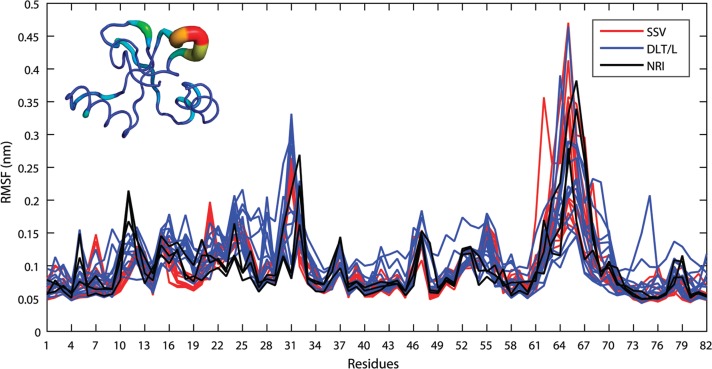
: root mean square fluctuation (RMSF) *per* residue of the inserted region. The lines are colored according to the defined motif, as indicated in the legend. The coil region comprising residues 61 to 70 displays the higher atomic fluctuations, as represented in the sausage plot at the upper left corner of the figure.

Next, all constructed 3D-structures were submitted to MD simulations in aqueous solution to examine their stability in a more realistic environment. All simulated models equilibrated after 20 ns of simulations during which the proteins underwent small conformational transitions due to adaptation to the solvent (Fig. 2A). Notably, the 62 a.a insertion portion remained stable during the entire simulations, thus indicating that no disturbance on its adjacent regions was introduced (Fig. 2B). Inspection of the atomic fluctuations of the insertion region during MD simulations revealed that structures belonging to the same subgroup presented similar dynamic behavior and deviations below 2.5 Å. The only exception was sequence LbrM.10.1560 that achieved a variation up to 3.0 Å. The coil region comprising residues 61 to 70 presented higher flexibility ~ 4.5 Å, which may attribute to its high degree of exposure to the solvent (Fig. 3). The dynamic properties of the active site were computed over the last five nanoseconds of the trajectories ensuring natural motions after equilibrium was achieved. In all simulations, the Zn^+2^ ion remained stacked within the active site with small variations in the average distances (2.2 ± 0.08 Å) between this ion and the Nε2 atoms from coordinating histidine residues H165, H169, and H235.

We wondered whether the sequence variations would affect structural and dynamic properties related to substrate recognition and binding. Firstly, we inspected the distribution of the electrostatic potential on the surface of leishmanolysins. Previously, it was shown that a large region of negative potential surrounded the active site of the available *L. major* leishmanolysin (Schlagenhauf et al. 1998). In that occasion the authors also reported no sufficient evidences to define the precise orientation of the protein on the promastigote membrane. Here, we initially performed a RMSD-based clustering of the trajectories and then submitted the central structure of the most populated cluster to electrostatic potential calculations.

**Fig. 4:**
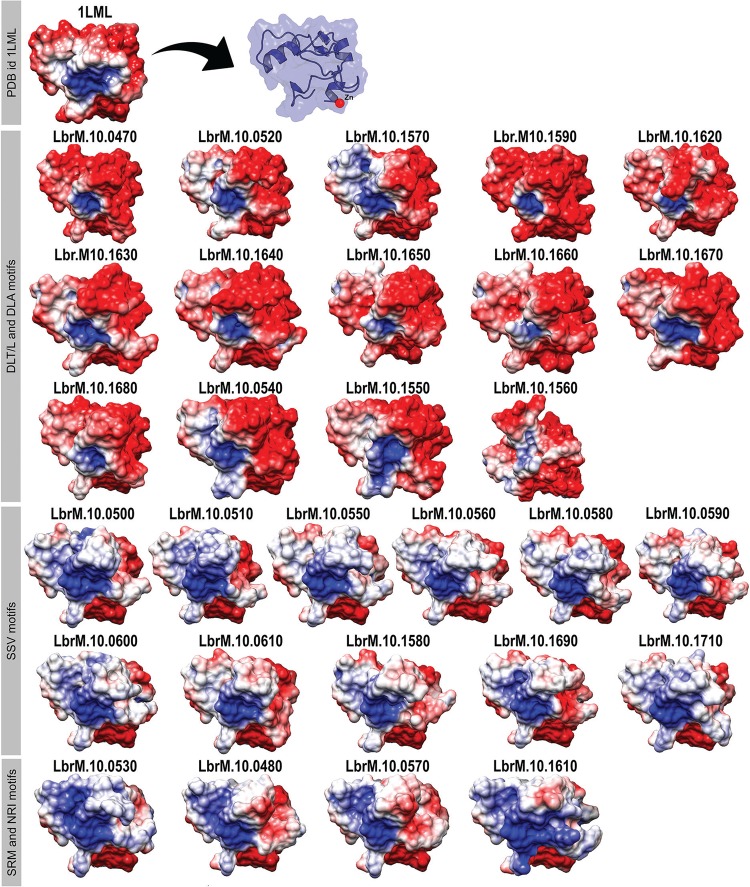
: differences in the surface electrostatic potential of the inserted region of M8 metalloprotease family of *Leishmania (Viannia) braziliensis*. The location of the zinc ion is indicated for the crystal structure (PDBid: 1LML). The molecular surface is colored according to electrostatic potential using the Chimera software; where red, white and blue correspond to acidic, neutral and basic potentials, respectively. The identities of the subgroups previously obtained are given in the left margin of the figure.

Fig. 4 shows the distribution of the electrostatic potential on the surface of each insertion and the binding site. We observed three distinct patterns of charge density that correlated well with the previously identified subgroups of sequences (Fig. 1A). Among the common features observed in all models, we noticed a conserved overall shape, except for the LbrM.10.1560 model. Also, as seen in the leishmanolysin available crystal structure (PDBid: 1LML), we noticed a small region predominantly positively charged in all structures.

The surface of leishmanolysins from the DLT/L subgroup presented large regions of negative electrostatic potential. These structures most resemble the distribution observed in the crystal molecule, thus corroborating the higher sequence identity between them when comparing with leishmanolysins from other subgroups.

This feature contrasts with the majority of neutral and basic areas found in the structures from the SSV subgroup. Proteins from the NRI subgroup share this pattern but they present a more positively charged region around helix H11 [adopting the nomenclature given in (Schlagenhauf et al. 1998)]. Therefore, these differences in the electrostatic profile besides corroborating the sequence-based analyses allow a clear separation among the three subgroups (Fig. 4).

**Fig. 5:**
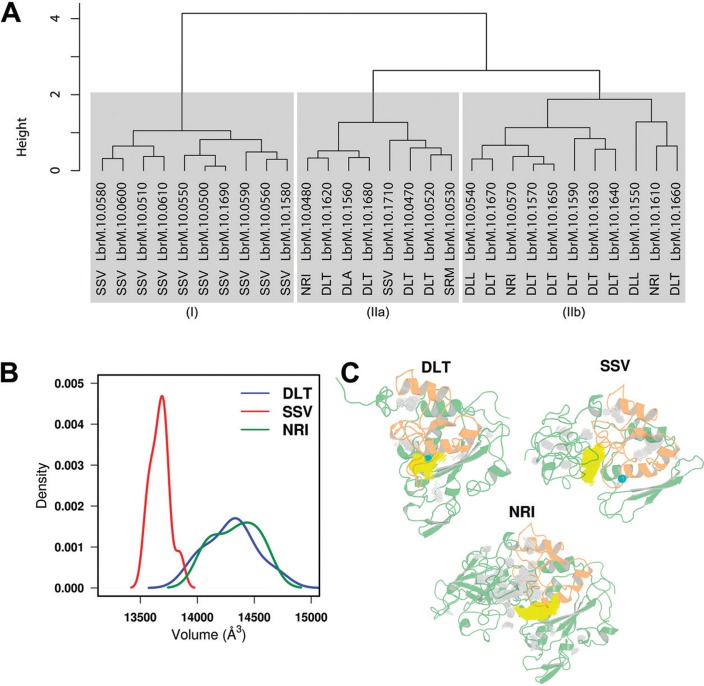
: (A) structure dendogram of the M8 metalloprotease family of *Leishmania (Viannia) braziliensis*. The scale on the left represents the height based on the Euclidean distance of standardised variables. Gray blocks highlight the formation of (I), (IIa) and (IIb) groups. The motif which identifies the structure is near the sequence name. (B) Distribution of the insertion region volumes calculated for each representative structure from distinct subgroups. Coloured as indicated in the legend. (C) Binding cavity analysis of the representative structures from the DLT, NRI and SSV subgroups. The insertion region is shown in orange while the largest cavity is highlighted in yellow. The catalytic zinc ion is represented as a cyan CPK sphere.

To gain insights into specific structural details of the active sites, we performed an integrative structural analysis using a selection of descriptors: volume, area, sphericity, effective radius and potential energy (Fig. 5). We obtained two major clusters of structures. The first group contained only sequences with SSV motif (n = 10). The second and largest cluster with 19 elements presented two subdivisions (IIa and IIb). The subcluster IIa arranged the structures containing DLT (n = 4), DLA (n = 1), NRI (n = 1), SRM (n = 1) and SSV (n = 1) motifs, whereas DLT (n = 7), DLL (n = 2) and NRI (n = 2) motifs were clustered in Group IIb. Curiously, proteins previously clustered in distinct groups (NRI and DLT/L) were placed in the same group according to their active sites structural parameters. The inspection of the volume of the insertion regions highlights such differences: while proteins from the DLT/L and NRI subgroups presented similar values, those from the SSV subgroup have minor volumes (Fig. 5B). As shown in Fig. 1B, enzymes from these two subgroups are evolutionary closer than those of the SSV subgroup. We also performed a detailed analysis of the binding site cavity using KVfinder (Oliveira et al. 2014). This analysis revealed that even though the SSV insertions are minor than those from the DLT/L and NRI subgroups, their binding cavities volumes are larger (Fig. 5C). Altogether, these last results reinforce the heterogeneity of leishmanolysins, since besides overall structural plasticity (Fig. 4), their regions involved in ligand binding (insertion region and active site) display considerable variability.

## DISCUSSION

*Leishmania* parasites present several adaptive peculiarities favoring their infection and survival. Leishmanolysins and lipophosphoglycan are the major components of the *Leishmania* cell surfaces. Previous data have shown that both are essential factors for *L. major* virulence (Pimenta et al. 1994). The target deletion of its seven leishmanolysin genes resulted in mutants 10-fold more sensitive to complement-mediated lysis. While reintroduction of only one gene (gp63 gene 1) partially restored the parasites ability to develop lesions, the presence of three genes (gp63 genes 1, 6 and 7) almost restored the wild-type phenotype (Joshi et al. 1998, Voth et al. 1998). In this occasion, it was suggested that different substrate specificities displayed by these proteins were an important feature to explain the importance of distinct leishmanolysin genes on the *L. major* cell surfaces (Joshi et al. 1998).

Here, we attempted to understand whether the high number of leishmanolysin genes in the *L. (V.) braziliensis* genome is correlated with higher structural plasticity, which ultimately allows efficient cleavage of a broader spectrum of substrates. From the comparison of 29 leishmanolysins sequences, we identified three subgroups displaying specific patches of residues along a 62 a.a insertion close to the active site. The heterogeneity is also observed at the structural level, as evidenced by the clear distinction between the electrostatic surfaces of each subgroup (Fig. 4). It is also worth mentioning that despite the considerable variation in the physicochemical characteristics of the insertions regions from different subgroups, they all presented a stable behavior during molecular dynamics simulations (Fig. 2).

These differences may influence the orientation of leishmanolysins along the membrane. Electrostatics indeed play a decisive role in protein orientation, as demonstrated for other proteins using experimental and theoretical methods (Zamarreño et al. 2012, Galassi et al. 2014). We hypothesize that the surface of *Leishmania* promastigotes is therefore composed of leishmanolysins adopting a broad range of orientations close to the membrane, therefore introducing an extra complexity that may favor the interactions with host cells. Also, the variations in the electrostatic potential in the region of the leishmanolysins active site certainly impact on their affinities for molecules with distinct charge distributions. Finally, this finding is also particularly important for the design of kinetic studies, since binding affinities measurements with immobilised proteins require their proper orientation (Talasaz et al. 2006).

We also investigated another level of structural diversity by comparing geometrical properties of the insertion regions and from the active sites. Interestingly, the separation of the structures obtained here differed from those previously found (Fig. 5A). Here, while the SSV subgroup present homogeneous geometries of their insertion regions, the enzymes with DLT/L and NRI motifs display more variable patterns. Regarding the presence of the leishmanolysins active site in the insertion region, we suggest the higher structural plasticity found in these two subgroups as an important aspect impacting on the ability of these enzymes to interact with molecules with varying volumes efficiently.

The comparison of the distributions of the insertion region volumes among distinct leishmanolysins confirms the higher heterogeneity found in enzymes with the DLT/L and NRI motifs (Fig. 5B). Here, we obtained broader distributions for proteins from these subgroups in comparison with those containing the SSV motif. However, a more detailed analysis of the binding cavities reveals that proteins with the SSV motif present a higher active site volume, which may enable their interactions with larger substrates (Fig. 5C). We stress here that a proper evaluation of protein cavities and ligand binding would require a more detailed study using Docking/Molecular dynamics simulations. Furthermore, the shape and size of these cavities are not static, since proteins are constantly undergoing structural rearrangements due to thermal motions.

Finally, in this study we were able to show that varying levels of heterogeneity are present in leishmanolysins from *L. (V.) braziliensis*, which probably confers to the parasite the ability to interact with various substrates, thus favoring the infection process and the interaction with the host’s immune system.
